# DBN Neural Network Model Combined with Meta-Analysis on the Curative Effect of Acupuncture and Massage

**DOI:** 10.1155/2022/8488167

**Published:** 2022-09-05

**Authors:** Xiujun Wang

**Affiliations:** School of Nutrition Clinic, The Third Hospital of Jinan Municipality, Jinan 250132, China

## Abstract

Acupuncture and massage are among the oldest medical treatments in China. During the acupuncture process, as well as the subsequent needle extraction process, there are differences in the acupuncture intensity, treatment duration, and acupuncture depth. For both medical treatments of acupuncture and massage, this article learns and analyzes a large amount of literature and applies DBN neural network method to build a human skeletal model to simulate and identify medical professional steps such as acupuncture therapy. The research results show that the recognition rate of DBN reaches 92.1% after the training of 1000 samples. After learning all the training samples, the DBN model achieved feature recognition accuracy of 96.4%, 97.68%, 96.66%, and 92.27% for the test samples of mixed needling process, needle insertion operation, needle extraction operation, and rotary needle handling process, respectively. The research in this article can contribute to the modernization of Chinese medicine by maximizing the simulation of the force on the human body when receiving needling and tui-na, as well as the clinical treatment effect.

## 1. Introduction

Acupuncture and massage, as one of the oldest medical means in China, have a long history of development and play a prominent role in China's medical field [1–3]. In China's medical field, people still habitually choose acupuncture and massage as treatments for some common problems such as acupoint blockage and local pain [4, 5]. Acupuncture is generally used to stimulate specific acupoints in the body to achieve therapeutic goals [6–8]. By stimulating the human body with sanyinjiao, Zusanli acupoints can affect blood circulation and stasis, Tianshu and Liangmen acupoints can promote digestion, etc. [1, 3, 9]. When the needle is inserted into the body, the body reacts to the stimulus and passes it on quickly. Then, the body responds to the action, and the pain-accumulating substances are redistributed, so that the pain is less likely to accumulate locally. After acupuncture enters the body, the immunity of the body can be mobilized, the body's response begins to strengthen, and the body becomes more combative, which can resist the invasion of external evil. Acupuncture can promote the improvement of self-healing ability. To be specific, when the human body is in a mild imbalance state, the body can be restored by self-adjustment. However, when the imbalance state is serious, external stimulation, such as acupuncture, is needed to stimulate the treatment, so as to improve the body's self-healing ability [10, 11].

The same message belongs to the external treatment of Traditional Chinese medicine, massage the specific parts of the body surface to achieve the purpose of regulating the viscera, dredging the meridians, promoting the normal operation of qi and blood, often used for the treatment of various pain, daily massage has the effect of relieving fatigue. Chinese medicine in the human body according to the meridians are acupoints with pushing, taking, lifting, kneading, and other techniques for treatment. Massage is a kind of non-drug natural therapy and physical therapy. The doctor uses his hands to act on the body surface of the patient, the injured part, the uncomfortable place, the specific acupoint, and the painful place, to achieve the curative effect of dredging the meridians, promoting qi and blood, helping the injury and relieving pain, dispelling evil and strengthening, and harmonizing Yin and Yang [12–15]. In addition, because massage is beneficial to circulation and metabolism, it has a positive therapeutic effect on patients with excessive weakness in general. In the long common field of traditional Chinese medicine, acupuncture and massage are usually combined to provide effective treatment for patients. However, in actual acupuncture and massage therapy for acupuncture needles of processes, stimulation, and subsequent needles pulled up in the process of the healer, stimulation treatment time, the problem such as the depth of the needles, these characteristics as the healer state each are not identical, there are differences, how to efficient, accurate to help doctors to solve these problems put forward the requirement to us.

At present, with the rapid development of science and technology and the improvement of computer networks, the application of virtual reality technology and neural network learning to some difficult training work, in reality, has been increasing [16–20]. In Xu et al. [21], they investigated the uncertainty of the dose-response relationship between acupuncture and major depression, including randomized controlled trials comparing acupuncture with sham acupuncture or antidepressants, data extraction, data quality, and bias risk assessment. Finally, a nonlinear meta-regression method with cubic spline restriction was used to study the dose-effect relationship between acupuncture courses and their effect on Hamilton Depression Scale (HAMD) score. Niu et al. [22] conducted a targeted study on the efficacy of acupuncture and massage, and their results showed that massage and acupuncture had good therapeutic effects on patients, but further regulation of the intensity of massage and acupuncture and other operations as needed. Their studies have shown the positive effect of acupuncture and massage on treatment, but in the process of acupuncture and massage, the strength of acupuncture, the depth of the needle, and the strength of massage need to be further standardized. Combining neural networks to optimize and improve problems is now a commonly used method. Belciug [23] proposed a potential deep learning method, network architecture automation. As the number of network architectures increases exponentially with the number of convolution layers in the network, a differential evolution algorithm is proposed to traverse the search space, generate a group of random individuals, and then mutate, recombine, and select. In each generation, individuals with the worst loss values are eliminated and replaced by more competitive individuals. The results show that the proposed method has a positive effect on medical image recognition. Liu et al. [24] proposed a medical image super-resolution algorithm (SR-DCNN) based on a neural network and using deconvolution, which can be used to recover high-resolution images from low-resolution images, especially for medical images with clinical significance in diagnosis, treatment, and research applications. Interestingly, Anilkumar et al. [25] detected leukemia by analyzing microscopic smear images to detect the presence of leukemia cells using an image processing-based method, and compared the efficacy of three optimization algorithms of stochastic gradient descent with momentum, root means square propagation, and adaptive moment estimation (ADAM) in all the classifications performed. Through the analysis of the above literature, it is shown that the application of deep learning neural networks in the medical field has been more and more extensive, and has achieved good results, which provides a solution to the problems related to acupuncture and massage. In addition, there are differences in some conditions of patients, even in the condition of the same influencing factor, there are differences in different patients and different ages. Therefore, we need to comprehensively compare the heterogeneity of the system, to better serve patients. Silvy et al. [26] used meta-analysis to explore the problem that cumulative meta-analysis can detect the presence or absence of treatment effects early in the treatment process, to avoid unnecessary and expensive new randomized trials. Katsanos et al. [27] analyzed and explained the relationship between statins and cerebral microbleeds (CMB) through meta-analysis, and analyzed and evaluated the association between the existence of CMB and the use of statins. Their study showed that meta-analysis showed excellent and stable performance in comparison and synthesis of research results. Compared with a single study, the integration of all acupuncture and message-related studies can more accurately standardize doctors and is conducive to exploring the consistency of evidence and differences among studies.

Therefore, in our research work, we focused on the acupuncture process of acupuncture, stimulation treatment, as well as the follow-up needle extraction process of the strength of the doctor, the time of stimulation treatment, the depth of acupuncture and other problems, the strength of massage and the corresponding different patients, and different ages of some different performance. We first by choosing DBN neural network for accurate identification of acupuncture and moxibustion in the process of operating characteristics, and through the meta-analysis of heterogeneity and multiple independent research statistic consistency check, to evaluate the effect of acupuncture and massage analysis for us, our research will contribute to the promotion and application of TCM acupuncture and massage.

## 2. Feasibility of DBN in the Evaluation of the Curative Effect of Acupuncture and Massage

In the context of the booming development of big data [28], due to the robustness of mesh meta-analysis and the accuracy of the analysis model, the computational modeling method driven by big data is more suitable for solving the optimal intervention scheme of acupuncture and massage for the treatment of various TCM bias.

### 2.1. Design of Multilayer Neural Network

In this article, the gradient descent method is used to train the multilayer neural network shown in the figure. The specific calculation process is as follows:

(1) $$\begin{array}{c} \Delta w=-\eta \frac{\delta E}{\delta w}\text{,} \end{array}$$

where ∆*w* is the adjustment value of the proportion of connection weight *w*; *E* is the error between actual results and predicted results; and *η* is the learning rate.

To update each parameter in the network model of acupuncture and massage efficacy, we need to define the loss function to compare the error between the actual result and the predicted result and update the hyperparameters through the partial derivative of the error function. The smooth *L*1 loss function is used in this article. The specific calculation process is as follows:

(2) $$\begin{array}{c} smooth_{L1}=\left\{\begin{array}{c} 0.5{\left(y-f\left(x\right)\right)}^{2},if\left|x\right|<1\\ \left|y-f\left(x\right)\right|-0.5,otherwise. \end{array}\right. \end{array}$$

where *y* is the actual result and *f* (*x*) is the predicted result.

In common machine learning, the gradient descent direction is the steepest. The smooth *L*1 loss function is minimized by iterating the calculated gradients in the network and updating the neural network model parameters in the reverse gradient direction so that only the minimum matching value of the gradient is used in each calculation. The calculation process is as follows:

(3) $$\begin{array}{c} g_{t}={\nabla_{\theta }}_{t-1}f\left(\theta_{t-1}\right)\text{,}\\ \Delta \theta_{t}=-\eta g_{t}\text{,} \end{array}$$

where *g*_*t*_ is the gradient and *θ* is the hyperparameter.

In addition, to accelerate the convergence speed of the model, the momentum optimization algorithm is used to update the parameters of the neural network model. The specific calculation process is as follows:

(4) $$\begin{array}{c} m_{t}=\mu m_{t-1}+g_{t}\text{,}\\ \Delta \theta_{t}=-\eta m_{t}\text{,} \end{array}$$

where *μ*is the momentum factor and *m*_*t*_ is the gradient descent loss function after the momentum optimization algorithm is added.

### 2.2. The Fundamentals of Deep Confidence Networks (DBN)

**Figure 1:**
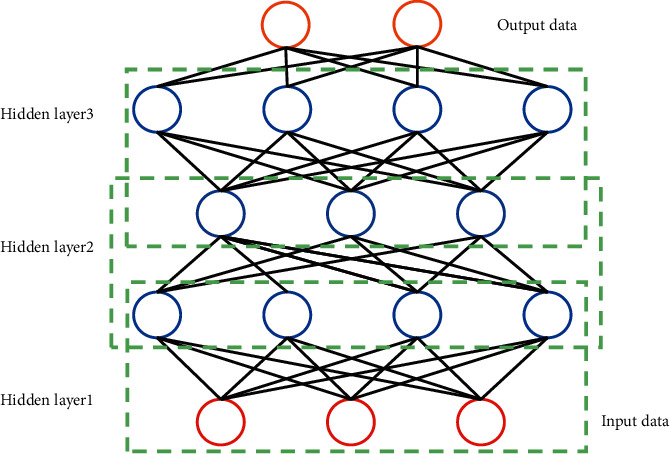
Flow chart of DBN network structure.

DBN updates and iterates the connection weight ratio in the deep belief network through the learning method of layer-by-layer neurons [29, 30]. Furthermore, the specific data of acupuncture and massage were extracted effectively by using the method of forwarding unsupervised layer-by-layer neuron training, and the therapeutic results of acupuncture and massage were transmitted back using supplementary supervision and fine-tuning to further calculate the optimal intervention scheme for treating various TCM biased constitution. The specific DBN network structure is shown in Figure 1.

In DBN, the neural network was used to generate a random evaluation of acupuncture and massage efficacy to help input acupuncture and massage data to complete probability distribution learning. We build our DBN model through Matlab software, and then we need to deal with basic information such as research objects, intervention measures, measurement indicators, treatment period, number of subjects, gender, physical fitness intervention measures, measurement indicators, and courses of treatment. It is used as the input data of DBN, and then it is continuously trained iteratively. Specifically, the energy function between the units that define the energy function of RBM, the hidden unit that calculates acupuncture and massage efficacy evaluation of each layer, and the units that visually output and input acupuncture and massage efficacy evaluation layer of each layer are described as follows:

(5) $$\begin{array}{c} E\left(v,h;\theta \right)=-\overset{n}{\underset{i=1}{\sum }}b_{i}v_{i}-\overset{n}{\underset{j=1}{\sum }}a_{i}h_{j}-\overset{n}{\underset{i=1}{\sum }}\overset{m}{\underset{j=1}{\sum }}v_{i}w_{ij}h_{j}\text{,} \end{array}$$

where *w* = (*w*_*ij*_), *iϵ*[1, *n*]*jϵ*[1, *m*] is the weight ratio matrix between the hidden unit of acupuncture and massage efficacy evaluation and the units of visual output and input acupuncture and massage efficacy evaluation layer; *a* = (*a*_*j*_), *jϵ*[1, *m*] is the bias of the implicit unit in the evaluation of the curative effect of acupuncture and massage; *b* = (*b*_*j*_), *iϵ*[1, *n*] is the bias of units in the evaluation layer of acupuncture and massage efficacy of visual output-input; *v* represents the input data of acupuncture and massage in DBN neural network; *h* is the output result of acupuncture and massage in DBN neural network; *i* is the neural layer; *j* is the *j*-th neuron in the *i*-th neural layer.

(6) $$\begin{array}{c} P\left(v,h,\theta \right)=\frac{1}{Z\left(\theta \right)}\exp\;\left(-E\left(v,h;\theta \right)\right)\text{,} \end{array}$$

where *P*(*v*, *h*; *θ*) defines the joint probability between the implicit unit of acupuncture and massage efficacy evaluation and the units of visual output-input acupuncture and massage efficacy evaluation layer based on the theory of thermodynamics.

(7) $$\begin{array}{c} Z\left(\theta \right)=\underset{v}{\sum }\underset{h}{\sum }\exp\;\left(-E\left(v,h;\theta \right)\right)\text{,} \end{array}$$

where *Z*(*θ*) is the normalized factor.

Based on the joint probability, the conditional probability and edge probability of the implicit unit of acupuncture and massage efficacy evaluation and the visual output-input of acupuncture and massage efficacy evaluation layer were further obtained. The specific calculation process is as follows:

(8) $$\begin{array}{c} P\left(h;\theta \right)=\frac{1}{Z\left(\theta \right)}\underset{v}{\sum }\exp\;\left(-E\left(v,h;\theta \right)\right)\text{,}\\ P\left(v;\theta \right)=\frac{1}{Z\left(\theta \right)}\underset{h}{\sum }\exp\;\left(-E\left(v,h;\theta \right)\right)\text{,} \end{array}$$

where *P*(*h*; *θ*) is the conditional probability of implicit unit of acupuncture and massage efficacy evaluation and visual output-input layer of acupuncture and massage efficacy evaluation; *P*(*v*; *θ*) is the edge probability of hidden unit of acupuncture and massage efficacy evaluation and visual output-input of acupuncture and massage efficacy evaluation layer.

In addition, the Sigmoid activation function is used as the activation function of the DBN neural network in this article. The specific calculation process is as follows:

(9) $$\begin{array}{c} \sigma \left(x\right)=\frac{1}{1+\exp\;\left(-x\right)}\text{.} \end{array}$$

Finally, since there is no connection between the implicit unit of acupuncture and massage efficacy evaluation and the visual output and input acupuncture and massage efficacy evaluation layer, the activation function between neurons in the DBN network is deduced. The specific calculation process is as follows:

(10) $$\begin{array}{c} P\left(\left.h_{j}=1\right|v;\theta \right)=\frac{1}{1+\exp\;\left(-a-\sum_{i}v_{i}w_{ij}\right)}\text{,}\\ P\left(\left.v_{i}=1\right|h;\theta \right)=\frac{1}{1+\exp\;\left(-b-\sum_{i}h_{i}w_{ij}\right)}\text{,} \end{array}$$

where *P*(*v*_*j*_=1|*h*; *θ*) is the conditional probability of implicit unit of acupuncture and massage efficacy evaluation and visual output-input layer of acupuncture and massage efficacy evaluation obtained through activation function sigmoid processing; *P*(*h*_*j*_=1|*v*; *θ*) is the edge probability of implicit unit of acupuncture and massage efficacy evaluation and visual output-input of acupuncture and massage efficacy evaluation layer obtained by activation function Sigmoid processing. The neural network model applied to evaluate the curative effect of acupuncture and massage; *x* represents the input data of layer *i* of acupuncture and massage curative effect; *w* represents the proportion of connection weight between layer *i* and layer *i* + 1 neurons; *b* represents bias; *z* represents the input data of neurons; and *a* represents the output result of calculation of the curative effect of acupuncture and massage by neurons. The input of neurons in layer *i* + 1 is the sum of the output result of acupuncture and massage efficacy of neurons in layer *i* multiplied by the connection weight proportion *w* and *b* bias.

The training process of DBN can be divided into two stages, namely pre-training and reverse fine-tuning. Firstly, the unsupervised greedy learning algorithm is used to train each RBM layer by layer, and the data feature information is transmitted layer by layer. The network parameters are initialized, and the initial connection weights and neuron bias are determined. Then, BP (Back Propagation) algorithm was used for pre-training. The obtained initial weights are fine-tuned from top to bottom, and supervised training is carried out to converge the model to the optimal solution, to determine the structure of the entire DBN network.

**Figure 2:**
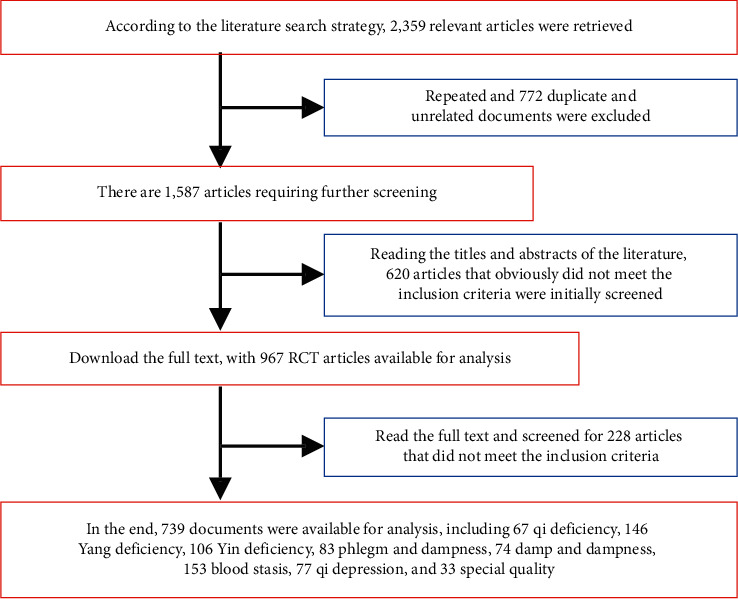
Database literature screening process.

Acupuncture and massage input data pretreatment. Classification software was used to classify the initially retrieved acupuncture and massage literature, and the literature that did not meet the standards was screened out. Further screening was conducted by reading the preliminarily obtained acupuncture and massage literature, and the number and reasons for the excluded literature were recorded at the same time. The main process is shown in Figure 2: the databases we searched are mainly divided into Chinese and English databases. The Chinese databases include CNKI Journal Full-text Database (CNKI), CNKI China Doctoral Dissertation Full-text Database, CNKI China Excellent Master's Thesis Full-text Database, Chinese Science and Technology Periodical Database-VIP Information Network (VIP), and Wanfang Database (WANFANG DATA). The English databases include CNKI Journal Full-text Database (CNKI), CNKI China Doctoral Dissertation Full-text Database, CNKI China Excellent Master's Degree Thesis Full-text Database, Chinese Science and Technology Periodical Database-VIP Information Network (VIP), and Wanfang Database (WANFANG DATA).

In conclusion, in the DBN neural network training process, forward propagation is trained by the input data matrix of acupuncture and massage, and the Gibbs sampling method is adopted to transfer the vector of visual output of RBM into the efficacy evaluation layer of acupuncture and massage to the implicit unit of efficacy evaluation of acupuncture and massage through the RBM network. Then based on the hidden unit of acupuncture and massage efficacy evaluation, the visual output-input acupuncture and massage efficacy evaluation layer was reconstructed and the parameters in DBN network were updated and iterated. Next, the implicit unit vector of acupuncture and massage efficacy evaluation of layer *i* obtained from training was used as the input vector of layer *i* + 1 RBM, and the next calculation was updated. After the loop reaches the maximum number of iterations, the DBN network stops computing and outputs the parameters.

## 3. Analysis and Discussion

A deep confidence network (DBN) is a kind of deep neural network based on a traditional constrained Boltzmann machine (RBM). DBN's training scheme is implemented layer by layer, which uses an input vector to calculate and infer the characteristics of the middle layer in the iterative training of each layer, and finally carry out the learning and promotion of the vector of the next layer based on the data and characteristics of the middle layer. In this article, a comprehensive and effective input database was established based on the evaluation of the efficacy of acupuncture and massage by a large number of scholars and researchers collected from the meta-analysis program established earlier. The database includes prior data on various types of patients' physical conditions and previous diagnoses. The analysis data in the database will be input into the established DBN neural network model for feature recognition training. And finally, form a specific program of acupuncture and massage according to the preliminary diagnosis results of patients. During the execution of acupuncture and massage therapy, DBN neural network model will provide precise and comprehensive assistance in strength for physiotherapists by learning the pressure data of standard acupuncture and massage, and help doctors judge the effectiveness of treatment. Finally, it provides an evaluation of the overall treatment plan and guidance for the follow-up treatment plan.

**Figure 3:**
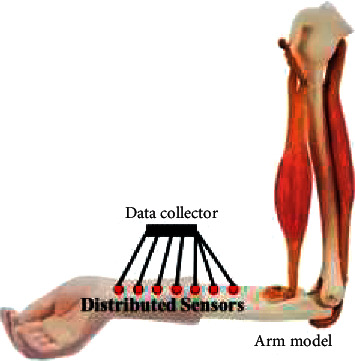
Schematic diagram of arm model.

Training the DBN model has practical guiding significance for factors such as acupuncture and massage strength in the process. In this section, we constructed a human skeleton model to simulate the stress of the actual human body receiving acupuncture and massage and the clinical effect of treatment to the greatest extent. To reduce the amount of calculation and improve accuracy, we focused the actual treatment on the arm part of the human body and carried out targeted acupuncture treatment for the patients. The trained DBN model can be further extended to other parts of patients and other acupoints. The specific composition of the model is shown in Figure 3. The components are silicone imitation of the human arm bone and similar density of flesh tissue model. According to the coordinates and depth of the actual acupoints in the arm, we set up multiple high-precision sensors at the corresponding positions in the arm model. Sensors are evenly distributed on the coordinate line of acupoint depth, and the collected pressure data are read and transmitted to the data collector for recording and feedback. The accuracy of the sensor and data collector has been corrected within a small error range.

**Figure 4:**
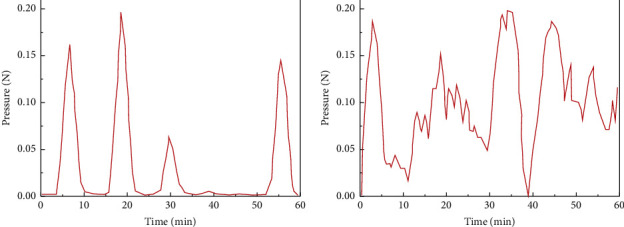
Instantaneous pressure signal diagram of needle: (a) lifting and inserting; (b) rotation.

Before constructing DBN neural network as a classifier for the specific process of acupuncture and moxibustion treatment for patients, we will first make the DBN model conduct appropriate special diagnosis recognition training for pressure transmission signals in each process of specific acupuncture and moxibustion therapy. For the accuracy and comprehensiveness of DBN model learning, we performed a complete treatment demonstration on the arm model using standard acupuncture treatment courses. The steps of the cadaver include needle insertion, extraction, and needle rotation. Figures 4(a) and 4(b), respectively, show the instantaneous pressure records of the sensor when the needle was inserted and pulled out and rotated in the patient's arm model. Among them, the amplitude of the signal waveform in Figure 4(a) is relatively regular, and the peak represents that we performed a complete needle insertion and extraction for the patient. Specifically, the rise of the crest is the characteristic signal fluctuation of needle insertion, while the decline of the crest in the response corresponds to needle extraction.  Figure 4(b) shows the waveform of the pressure signal caused by needle rotation. It is worth noting that the needle rotation is rotated after puncturing the acupoint, so the waveform presented is a discontinuous stimulus signal composed of more pulse signals with smaller amplitude.

We now summarize the input and output of the feature recognition classifier adapted to DBN neural network architecture for the complete arm acupuncture treatment process. For example, in the collection of experimental data, it is found that the main signal recognition feature of needle insertion, extraction, and rotation is different in the waveform diagram in Figure 4. Specifically, we found that the transient pressure value caused by the needle insertion operation was positive, that is, the pressure at the next moment was greater than that at the last moment, which could be explained by the increased tissue penetration resistance encountered by the needle after conducting human skin tissue. On the contrary, the transient pressure value caused by the needle pulling out operation is negative, that is, the pressure at the next moment is less than that at the last moment, which is caused by the small diameter of the tip. For the wave signal of needle rotation, the transient change of pressure is positive and negative, but it is not easy to determine and identify the classification of a single wave peak, so we selected the 60-minute acupuncture treatment process for the overall training and recognition classification in the follow-up. Therefore, for the input data vector of the DBN model, we choose the value of transient change of pressure.

**Figure 5:**
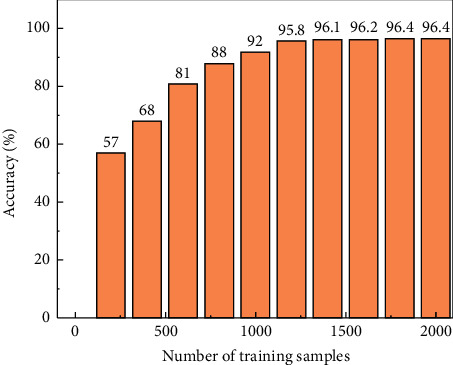
Relationship between identification accuracy and sample number of DBN neural network model.

To ensure the reliability, accuracy, and comprehensiveness of the results, we judge the convergence of the trained DBN neural network model. DBN was learned from the signal data of the previous standard acupuncture practices of professional physicians. A total of 2115 sets of needle insertion, extraction, and rotation data were obtained for training. There were 907 mixed tests of needle insertion, extraction, and rotation.  Figure 5 shows the trend of feature recognition accuracy of the trained DBN neural network model with the variable of several training samples. It was observed that DBN achieved relatively accurate recognition after learning 1000 training sample species, and its recognition rate reached 92.1%. Then the rising trend of accuracy becomes slow, which indicates that the input of the DBN model and the selection of the input quantity of feature recognition are reasonable. After learning all the training samples, DBN's feature recognition accuracy of the mixed acupuncture process of the test samples reached 96.4%. The reliability of targeted acupuncture therapy has been established to assist physicians.

**Figure 6:**
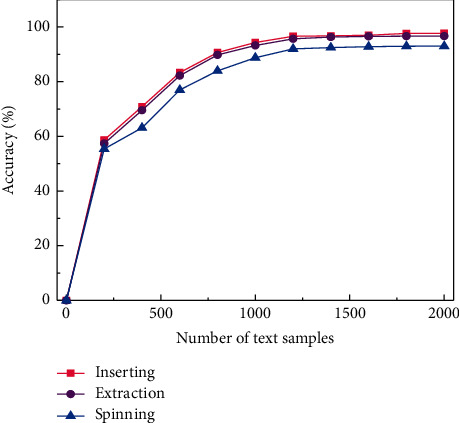
The trend of DBN-based feature recognition rate as a function of the number of tests.

Further, Figure 6 shows the trend of the feature recognition accuracy of the DBN neural network model for the three steps of insertion, extraction, and rotation as a function of the number of tests. It can be seen that consistent with the accuracy of sample training, the accuracy of test training also increases with the increase of the number of tests, and the overall accuracy is close to that of sample training. When the number of pieces of training reaches 1000, the three-step accuracy rates are all close to 90% accuracy, and then the accuracy rate rises slowly. After reaching 2000 training times, the recognition accuracy of needle insertion and extraction steps reached 97.68% and 96.66%, respectively, and the recognition accuracy of needle rotation therapy was 92.97%.

Subsequently, we made statistics on the accuracy of the prediction of test data of acupuncture steps based on experimental data and the DBN neural network model at the end of the meta-analysis comprehensive training.

## 4. Results

**Figure 7:**
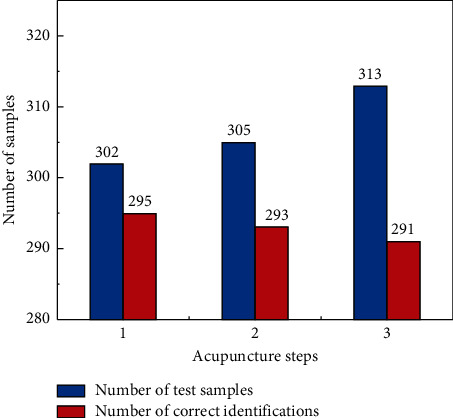
Total sample number and correct sample number of acupuncture steps identified by DBN neural network model.

As shown in Figure 7, it was observed that the recognition accuracy of the trained DBN model for needle insertion and extraction operations reached 97.68% and 96.66%, respectively. The accuracy of DBN in identifying the needle rotation treatment process was 92.97%. The results are similar to the number of training samples and test samples for the three steps of acupuncture. Therefore, we can observe that the trained DBN neural network has excellent recognition accuracy between needle insertion and withdrawal with relatively regular pressure signals and large changes in instantaneous pressure values. For the needle rotation action with more changes in the amplitude of the pressure signal, lower amplitude, and more pulse signals, the recognition accuracy of the DBN model is slightly reduced, which is caused by the complexity of the signal. Therefore, we will conduct further research on the distribution position of the pressure sensor and the noise error generated in the future to reduce the influence of the noise data of the input signal itself on the DBN model.

**Figure 8:**
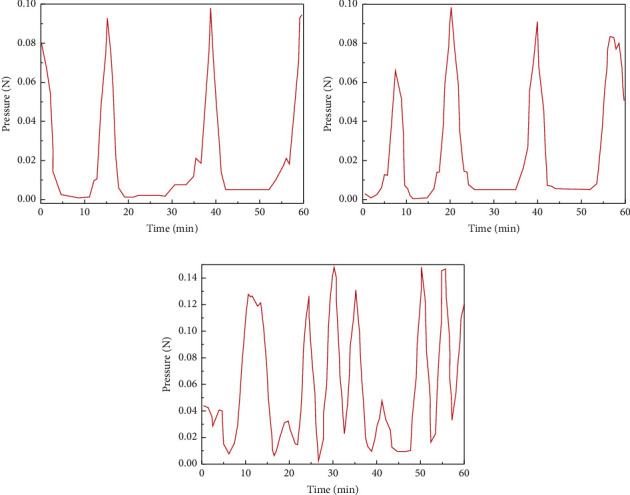
Clinical tests of DBN neural network model to identify acupuncture steps: (a) inserting; (b) extraction; (c) spinning.

Finally, to highlight the help of a trained DBN neural network model for conventional acupuncture treatment, professional physicians randomly performed three typical procedures in clinical practice and identified them using the DBN model.  Figure 8 shows the pressure inputs for three typical operations, which DBN identifies as needle insertion, pull out, and rotation operations. In addition, we have built in the appropriate range of the needle insertion and rotation depth, to detect and alarm the optimal needle treatment depth, and to help acupuncturists better accurately targeted treatment for patients.

## 5. Conclusion

Acupuncture and massage are one of the oldest medical means in China, with a long history of development and an outstanding position in China's medical field. However, in the actual acupuncture and massage treatment, there are differences in the strength, time of stimulation, and depth of acupuncture in the acupuncture process, as well as in the subsequent needle pulling process. Aiming at the basic acupuncture process in acupuncture and massage, DBN neural network model was established based on a large amount of information collected from the meta-analysis database, and the acupuncture techniques and steps of professional physicians were deeply learned. In addition, we have built in the DBN neural network model the appropriate interval range for the depth of needle insertion and rotation, to detect and alarm the optimal needle treatment depth, and to help acupuncturists better accurately target patients for treatment. This is expected to provide powerful help for more efficient clinical diagnosis and treatment of patients by acupuncture and massage physicians in the future.

1. This article establishes a comprehensive and effective input database based on the evaluation of therapeutic effects of acupuncture and massage by a large number of scholars and researchers collected from the meta-analysis scheme established earlier. At the same time, we constructed a human skeleton model to simulate the actual human body's stress and the clinical effect of acupuncture and massage. The two parts of data are the learning and training input data of the DBN neural network model.
2. We performed a complete treatment demonstration on the arm model using standard acupuncture treatment courses. The steps of the cadaver include needle insertion, extraction, and needle rotation. It was observed that the recognition rate of DBN reached 92.1% after learning 1000 training samples. After learning all the training samples, DBN's feature recognition accuracy of the mixed acupuncture process of the test samples reached 96.4%.
3. The accuracy of the prediction of test data of acupuncture steps was statistically analyzed based on the DBN neural network model after the comprehensive training of experimental data and meta-analysis. It was observed that the recognition accuracy of the trained DBN model for needle insertion and extraction was 97.68% and 96.66%, respectively. The accuracy of DBN in identifying the needle rotation treatment process was 92.97%.

Because of the complexity of the signal, the recognition accuracy of DBN for needle rotation is slightly reduced. Therefore, we will further study the distribution position of the pressure sensor and the noise error generated in the future, to reduce the impact of the noise data of the input signal itself on the DBN model. However, the application of the DBN neural network model in this field is expected to provide powerful help for acupuncture and massage physicians to carry out more efficient clinical diagnoses and treatment for patients in the future.

## Data Availability

The dataset can be accessed upon request.
